# Sepsis results in early cholesterol and steroidogenesis pathway alterations

**DOI:** 10.1186/2197-425X-3-S1-A978

**Published:** 2015-10-01

**Authors:** W Khaliq, DT Andreis, M Singer

**Affiliations:** Bloomsbury Institute of Intensive Care Medicine, University College London, London, United Kingdom; Dipartimento di Fisiopatologia Medico-Chirurgica e dei Trapianti, Università degli Studi di Milano, Milan, Italy

## Introduction

Both steroid and sex hormones regulate multiple physiological functions including the stress response, fluid retention, metabolism and reproduction. They are synthesized from cholesterol via the precursor steroid pregnenolone. We previously reported early falls in HDL and LDL cholesterol levels in a fluid-resuscitated rat model of faecal peritonitis [1] and these could prognosticate as early as 6 h post-insult [2]. Pregnenolone may be converted to either (i) progesterone, corticosterone and then aldosterone via a common pathway or (ii) to dehydroepiandrosterone (DHEA), the precursor to testosterone and estradiol.

## Objectives

Using our 72 h rat model of faecal peritonitis, we sought to investigate the relationship between hypocholesterolaemia and abnormalities in the steroidogenesis pathways in sepsis.

## Methods

Awake, instrumented yet fully mobile male Wistar rats (325 ± 15g) received an i.p. injection of 4µl/g faecal slurry. Fluid resuscitation (50:50 mix of 5% glucose/Hartmann's; 10 ml/kg/h) was commenced at 2 h. At 6 h, an echo-measured heart rate cut-off of 460 bpm was used to classify animals into predicted survivors or non-survivors. Blood samples were taken concurrently for measurement of the lipid profile (enzymatic colorimetric analysis) and plasma levels of progresterone, corticosterone, aldosterone, DHEA, testosterone and estradiol (ELISA). Control animals were treated identically except for slurry injection. Results were analysed using two-way ANOVA and post-hoc testing and considered statistically significant when p < 0.05.

## Results

At 6 h septic animals had significant hypocholesterolaemia, the magnitude of which was significantly greater in predicted non-survivors. Levels in the progesterone-corticosterone-aldosterone steroidogenesis pathway were all elevated (p < 0.05 vs controls), whereas DHEA-testosterone-estradiol steroidogenesis pathway levels were all subnormal (Table 1).

**Table 1:** Table 1

	Control (n = 6)	Predicted survival (n = 6)	Predicted non-survival (n = 6)
HDL cholesterol (mmol/L)	1.03 ± 0.07	0.88 ± 0.04 ^a^	0.73 ± 0.07 ^a, b^
Progesterone (ng/mL)	31.1 ± 3.5	38.3 ± 5.2	41.6 ± 3.7 ^a^
Corticosterone (pg/mL)	231 ± 36	661 ± 121 ^a^	719 ± 87 ^a^
Aldosterone (pg/mL)	86 ± 8	172 ± 24 ^a^	619 ± 109 ^a, b^
DHEA (pg/mL)	138 ± 13	109 ± 21 ^a^	66 ± 13 ^a, b^
Testosterone (pg/mL)	156 ± 14	107 ± 8 ^a^	100 ± 7 ^a^
17β-Estradiol (pg/mL)	84 ± 6	71 ± 8	69 ± 9
***Data shown as median ± SE;*** ^***a***^ ***p < 0.05 versus control,*** ^***b***^ ***p < 0.05 versus survivors***			

## Conclusions

Sepsis resulted in significant early reductions in cholesterol levels. This reduction may be due to increased cholesterol utilization for preferential steroidogenesis in the progesterone-corticosterone-aldosterone pathway, at the expense of DHEA-testosterone-estradiol steroidogenesis. This relationship has not, to our knowledge, been previously described in sepsis and warrants further investigation.

## Grant Acknowledgment

ESICM Basic Science Award, Intensive Care Foundation (UK), NIHR
